# Supplementary material for: The pyrethroid insecticide deltamethrin disrupts neuropeptide and monoamine signaling pathways in the gastrointestinal tract
Source: bioRxiv. 2024 Dec 17:2024.12.14.628386. Preprint. [Version 1] doi: 10.1101/2024.12.14.628386 (PMC11702531; doi:10.1101/2024.12.14.628386)
Supplement: Supplement 1 [file media-1.pdf]

The pyrethroid insecticide deltamethrin disrupts neuropeptide and monoamine signaling pathways in the gastrointestinal tract

Alexandria C. White[1,2], Ian N. Krout[1,2], Sabra Mouhi[1], Jianjun Chang[1], Sean D. Kelly[1], W. Michael Caudle[3], Timothy R. Sampson*[1,2]

[1]Dept of Cell Biology; Emory University School of Medicine; Atlanta GA USA 30329

[2]Aligning Science Across Parkinson's (ASAP) Collaborative Research Network; Chevy Chase MD 20815

[3]Gangarosa Dept of Environmental Health, Rollins School of Public Health; Emory University; Atlanta GA 30329

*To whom correspondence should be addressed: trsamps@emory.edu

Supplementary Information

Supplementary Figure S1
Supplementary Figure S2
Supplementary Table S1

Supplementary Figure S1

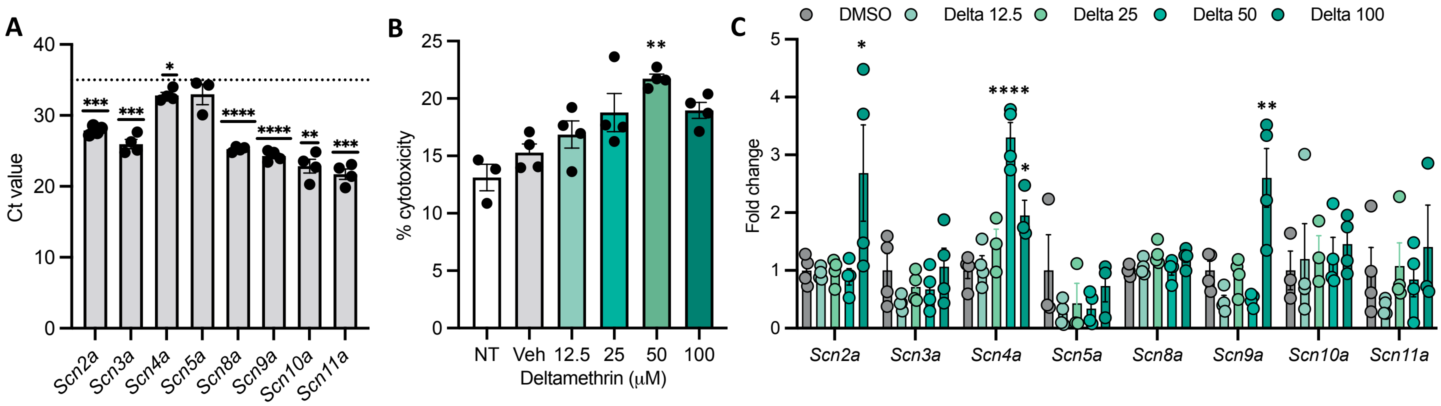

Supplementary Fig. S1. STC-1 cells express deltamethrin-sensitive voltage-gated sodium channels. A Ct values of each voltage-gated sodium channel (VGSC) subtype expressed in STC-1 cells. **B** Quantification of % cytotoxicity by lactate dehydrogenase (LDH) detection assay from STC-1 cells treated with 0, 12.5, 25, 50, or 100 μM deltamethrin for 24h. **C** Fold change of different VGSC subtypes from STC-1 cells treated with 0, 12.5, 25, 50, or 100 μM deltamethrin for 24h, as determined by qPCR. **A-C** All data points represent averages of technical duplicates from an individual well (n = 3-4). Data are depicted as mean $\pm$ SEM and compared by ordinary one-way ANOVA with Dunnett's multiple comparisons tests (**B, C**), one-sample t-tests against a Ct cutoff value of 35 (**A**), represented by a dashed line. *p<0.05, **p< 0.005, ****p<0.0001.

Supplementary Figure S2

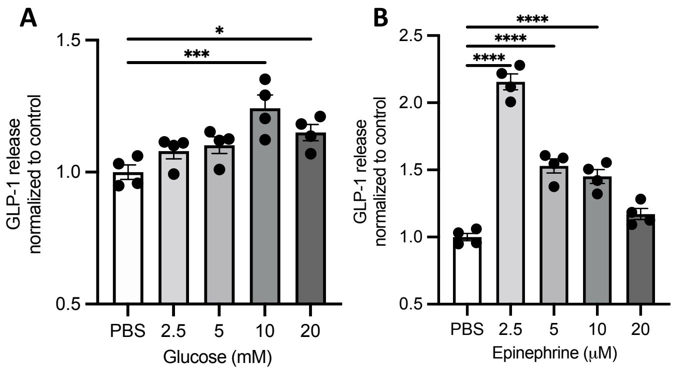

Supplementary Fig. S2. Glucose and epinephrine evoke GLP-1 release in STC-2 cells. A Quantification of GLP-1 release by ELISA from STC-1 cells treated with 0, 2.5, 5, 10, or 20 mM glucose for 1h. **B** Quantification of GLP-1 release by ELISA from STC-1 cells after treatment with 0, 2.5, 5, 10, or 20 μM epinephrine for 1h. **A-B** All data points represent averages of technical duplicates from individual samples where n = 3-4 per group. Data are depicted as mean $\pm$ SEM and compared by ordinary one-way ANOVA with Dunnett's multiple comparisons test comparing each group to the control (**A-B**). *$p<0.05$, ***$p<0.001$, ****$p<0.0001$.

Supplementary Table 1

RESOURCE TYPE	RESOURCE NAME	SOURCE	IDENTIFIER	NEW/REUSE
Protocol	STC-1 cell culture	protocols.io	dx.doi.org/10.17504/protocols.io.j8nlk98kwv5r/v1	reuse
Protocol	Dye-based intestinal motility	protocols.io	dx.doi.org/10.17504/protocols.io.eq2ly6pjwgx9/v1	new
Protocol	Total GI transit assay	protocols.io	dx.doi.org/10.17504/protocols.io.14egn9676l5d/v1	reuse
Protocol	Fecal Output	protocols.io	dx.doi.org/10.17504/protocols.io.rm7vzj3j5lx1/v1	reuse
Protocol	LDH cytotoxicity assay	protocols.io	dx.doi.org/10.17504/protocols.io.261ger51yl47/v1	reuse
Protocol	GLP-1 ELISA assay	protocols.io	dx.doi.org/10.17504/protocols.io.j8nlkokm6v5r/v1	reuse
Protocol	Western blot	protocols.io	dx.doi.org/10.17504/protocols.io.kxygxwyxkv8j/v1	reuse
Protocol	RNA extraction	protocols.io	dx.doi.org/10.17504/protocols.io.e6nvwdyn7lmk/v1	reuse
Protocol	cDNA synthesis	protocols.io	dx.doi.org/10.17504/protocols.io.14egn9676l5d/v1	reuse
Protocol	RT qPCR	protocols.io	dx.doi.org/10.17504/protocols.io.36wgqdn1ovk5/v1	new
Experimental model: Cell line	STC-1 cell line	ATCC	Cat#: CRL-3254	reuse
Experimental model: Organism/strain	C57BL/6J mice	Jax	RRID: IMSR_JAX:000664	reuse
Chemical, peptide, or recombinant protein	Deltamethrin	Chem Service	Cat#: N-11579-250MG	reuse
Chemical, peptide, or recombinant protein	Glucose	Sigma	Cat#: G8270-1KG	reuse
Chemical, peptide, or recombinant protein	Epinephrine	Sigma	Cat#: E4250-1G	reuse

Chemical, peptide, or recombinant protein	Carmine red dye	Sigma	Cat#: C1022	reuse
Chemical, peptide, or recombinant protein	Ensure Original mixed-meal nutrient drink	Abbott Pharmaceuticals	SKU#: 57243	reuse
Critical commercial assay	GLP-1 ELISA kit	Millipore	Cat#: EGLP-35K	reuse
Critical commercial assay	LDH cytotoxicity kit	ProMega	Cat#: G1780	reuse
Critical commercial assay	U-PLEX Metabolic Hormones Combo 1 for mouse	MSD	Cat#: K15306K-2	reuse
Oligonucleotide	*Tph1* qPCR primer forward	IDT	CCATCTTCCGAGAGCTAAACAAA	new
Oligonucleotide	*Tph1* qPCR primer reverse	IDT	TCTTCCCGATAGCCACAGTATT	new
Oligonucleotide	*Tph2* qPCR primer forward	IDT	TCGAAATCTTCGTGGACTGCG	new
Oligonucleotide	*Tph2* qPCR primer reverse	IDT	CGGATTCAGGGTCACAATGGT	new
Oligonucleotide	*Vmat1* qPCR primer forward	IDT	GTCCCGGAAGCTGGTGTTG	new
Oligonucleotide	*Vmat1* qPCR primer reverse	IDT	ACAGTGAGCAGCATATTGTCC	new
Oligonucleotide	*Vmat2* qPCR primer forward	IDT	CGCAAGCTGATCCTGTTCATC	new
Oligonucleotide	*Vmat2* qPCR primer reverse	IDT	ACGACGGTGAGCAGCATGT	new
Oligonucleotide	*Slc6a4* qPCR primer forward	IDT	TATCCAATGGGTACTCCGCAG	new
Oligonucleotide	*Slc6a4* qPCR primer reverse	IDT	CCGTTCCCCTTGGTGAATCT	new
Oligonucleotide	*Ddc* qPCR primer forward	IDT	TAGCTGACTATCTGGATGGCAT	new
Oligonucleotide	*Ddc* qPCR primer reverse	IDT	GTCCTCGTATGTTTCTGGCTC	new
Oligonucleotide	*Maoa* qPCR primer forward	IDT	GTGAATGTCAATGAGCGTCTAGT	new
Oligonucleotide	*Maoa* qPCR primer reverse	IDT	TCAACAGGGATCTCTTTTCCCA	new
Oligonucleotide	*Comt* qPCR primer forward	IDT	CTGGGGGTTGGTGGCTATTG	new
Oligonucleotide	*Comt* qPCR primer reverse	IDT	CCCACTCCTTCTCTGAGCAG	new

Oligonucleotide	*Glp1r* qPCR primer forward	IDT	TCAGAGACGG TGCAGAAATGG	new
Oligonucleotide	*Glp1r* qPCR primer reverse	IDT	ATCAAAGGTCC GGTTGCAGAA	new
Oligonucleotide	*Gapdh* qPCR primer forward	IDT	TGGCCTTCCGT GTTCCTA	new
Oligonucleotide	*Gapdh* qPCR primer reverse	IDT	GAGTTGCTGTT GAAGTCGCA	new
